# Intravitreal Injection of Splice-switching Oligonucleotides to Manipulate Splicing in Retinal Cells

**DOI:** 10.1038/mtna.2015.24

**Published:** 2015-09-01

**Authors:** Xavier Gérard, Isabelle Perrault, Arnold Munnich, Josseline Kaplan, Jean-Michel Rozet

**Affiliations:** 1 Laboratory of Genetics in Ophthalmology, Inserm UMR1163, Institut Imagine, Université Paris Descartes Sorbonne Paris Cité, Hôpital Necker, Paris, France

**Keywords:** CEP290, intravitreal injection, Leber congenital amaurosis, 2′OMePS antisense oligonucleotide, retinal dystrophies, splice-switching therapy

## Abstract

Leber congenital amaurosis is a severe hereditary retinal dystrophy responsible for neonatal blindness. The most common disease-causing mutation (c.2991+1655A>G; 10–15%) creates a strong splice donor site that leads to insertion of a cryptic exon encoding a premature stop codon. Recently, we reported that splice-switching oligonucleotides (SSO) allow skipping of the mutant cryptic exon and the restoration of ciliation in fibroblasts of affected patients, supporting the feasibility of a SSO-mediated exon skipping strategy to correct the aberrant splicing. Here, we present data in the wild-type mouse, which demonstrate that intravitreal administration of 2'-OMePS-SSO allows selective alteration of *Cep290* splicing in retinal cells, including photoreceptors as shown by successful alteration of *Abca4* splicing using the same approach. We show that both SSOs and *Cep290* skipped mRNA were detectable for at least 1 month and that intravitreal administration of oligonucleotides did not provoke any serious adverse event. These data suggest that intravitreal injections of SSO should be considered to bypass protein truncation resulting from the c.2991+1655A>G mutation as well as other truncating mutations in genes which like *CEP290* or *ABCA4* have a mRNA size that exceed cargo capacities of US Food and Drug Administration (FDA)-approved adeno-associated virus (AAV)-vectors, thus hampering gene augmentation therapy.

## Introduction

Retinal diseases are a leading cause of incurable severe visual dysfunction worldwide and hence a major public health problem. Leber congenital amaurosis (LCA, MIM204000) is the earliest and most severe of these diseases and a leading cause of blindness in childhood. It is characterized by major clinical, genetic, and physiopathological heterogeneity with non syndromic and syndromic forms, variable visual outcomes, and more than 45 disease genes with variable inheritance, pattern of expression, and retinal function.^1^ The safety and efficacy of AAV-based gene replacement therapy in LCA patients harboring *RPE65* mutations have paved the way for treating retinal diseases.^2,3,4^ However, FDA-approved AAV vector genomes are limited in size. The 7.9 kb *CEP290* cDNA are currently not amenable to AAV-based gene therapy.

*CEP290* encodes a 290 KDa centrosomal protein, which has an essential role in the development and maintenance of primary and motile cilia.^5,6,7^ *CEP290* mutations cause both nonsyndromic LCA and syndromic forms with renal, kidney, neural tube, central nervous systems, and/or bone involvement.^8^ Over 100 unique *CEP290* mutations are reported which include a recurrent deep intronic mutation underlying 10–15% of nonsyndromic LCA cases (c.2991+1655A>G).^8,9,10,11^ This mutation is located in intron 26 where it activates a cryptic splice donor site downstream of a strong acceptor splice site. The transcription of the mutant allele gives rise to a mRNA retaining a 128 bp intronic sequence encoding a premature termination codon along with low levels of the wildtype transcript. Recently, we reported 2'-O-methyl-phosphorothioate (2'-OMePS) splice switching oligonucleotide (SSO) sequences which allowed correcting the aberrant splicing and ciliation in fibroblasts from patients harboring the mutation.^12^ Delivery of 2'-OMePS SSOs to the retina is challenging. Approaches of systematically and topically delivered oligonucleotides have not been successful so far to reach intraocular tissues, probably due to the blood–retina barrier,^13^ and the impermeable nature of the cornea,^14^ respectively. Intraocular administration of SSO to target retinal cells has not been reported to our knowledge. A transgenic mouse harboring the human *CEP290* mutant intron has been produced which does not recapitulate the human molecular and clinical phenotypes.^15^ Here, studying the wild-type mouse, we report selective skipping of *Cep290* premessenger RNA sequences using a unique intravitreal (iv) injection of SSO. We show that both the SSO and skipped mRNA were detectable for at least 1 month and that iv administration of oligonucleotides did not provoke any serious adverse event.

## Results

**Figure 1:**
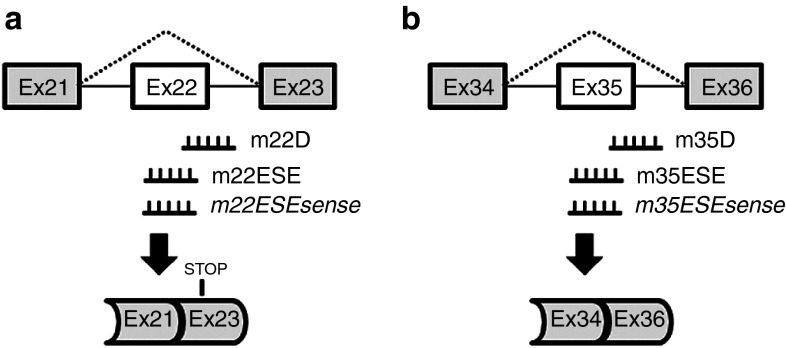
**SSO-mediated splicing alteration of *Cep290* pre-mRNA**. Schematic organization of target *Cep290* pre-mRNA structure, localization of oligonucleotides, and expected skipping. Contrary to m22ESEsense and m35ESEsense oligonucleotides, the m22D and m22ESE, and the m35ESE and m35D SSOs have been designed to skip exons 22 (**a**) and 35 (**b**), respectively. Skipping of exon 22 is expected to lead to the introduction of a premature stop codon in exon 23 whereas that of exon 35 is predicted to preserve the reading frame.

We designed SSOs to skip exon 22 (disruption of the reading frame) and exon 35 (preservation of the reading frame) of the mouse *Cep290* wild-type pre-mRNA, respectively. SSO sequences were designed using the m-fold and ESEfinder programs as described previously.^12^ For each of the two exons, we produced a set of three 2′-OMePS oligonucleotides. Each set included one SSO targeting the donor splice site (m22D and m35D), one SSO recognizing an exonic splice enhancer (m22ESE and m35ESE), and one control oligonucleotide (m22ESEsense and m35ESEsense, *i.e.*, sense versions of m22ESE, m35ESE SSOs, respectively; **Figure 1**).

We assessed SSO-mediated skipping in mouse NIH3T3 fibroblasts as described previously (**Supplementary Figure S1**).^12^ Transfection of the cells with the SSOs but not the control oligonucleotides resulted in the production of a mRNA lacking the targeted exon, and a significant reduction in wild-type mRNA and protein abundance, as determined by Sanger sequencing of reverse transcription polymerase chain reaction (RT-PCR) products, RT-qPCR, and Western blot analysis of immune-precipitated cep290, respectively (**Supplementary Figure S2**).

**Figure 2:**
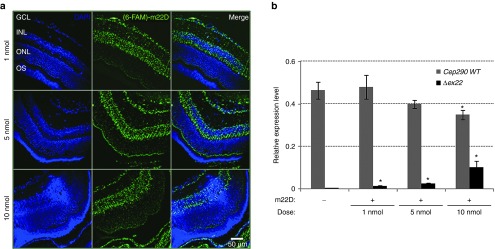
**Distribution and dose-dependent SSO-mediated *Cep290* splicing at 2 dpi**. (**a**) Confocal microscopy images of retina from C57BL/6J mouse eyes injected with 1 μl of saline solution containing 1 nmole, 5 nmoles, or 10 nmoles of (6-FAM)-m22D SSO showing fluorescent signals through-out all retinal layers with a maximum staining of the ONL at 10 nmoles. Nuclei were labeled using DAPI (blue). GCL, ganglion cell layer; INL, inner nuclear layer; ONL, outer nuclear layer; OS, outer segment of photoreceptors. Scale bar: 50 µm. (**b**) Relative expression of wild-type (*WT*, gray bars) and skipped (*Δex22*, black bars) *Cep290* mRNA in retina from uninjected (-) and injected (+; same conditions as in **a**) as determined by RT-qPCR using *Tbp* and *Hprt1* genes as reference. The error bars represent the standard error of the mean derived from three independent experiments from at least three eyes (*P < 0.05). Dpi, days postinjection.

These data supported the efficiency and specificity of our SSOs to mediate *Cep290* exon skipping *in vitro*. We thus assessed the retinal distribution of SSOs and splicing alteration using the iv delivery route. To this aim, we examined retinal sections and *Cep290* mRNA from 8-week-old C57BL/6J mice eyes at day 2 following a unique and unilateral iv injection of variable doses (1, 5, 10 nmoles) of a fluorescently-labeled (6-FAM)-m22D SSO in saline solution (NaCl 9 g/l; pH = 8.7; 290 mOsm/kg); fellow untreated eyes were used as controls. Confocal microscopy analysis of retinal sections from injected eyes detected panretinal fluorescent signals (**Figure 2a**; **Supplementary Figure S3**). Signals in the photoreceptor cell layer were maximal at the highest SSO dose (10 nmoles; **Figure 2a**). RT-qPCR analysis of retinal mRNA identified a transcript lacking exon 22 which in abundance increased with SSO concentrations (**Figure 2b**). No skipped *Cep290* mRNA and no fluorescent signals were observed in uninjected fellow eyes (**Figure 2b**; **Supplementary Figure S3**), giving support to the view that iv administration of 2'-OMePS SSO allows manipulating the splicing of the *Cep290* pre-mRNA in retinal cells, in a dose-dependent manner.

**Figure 3:**
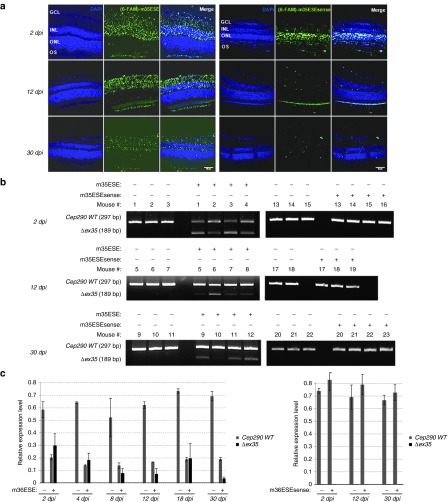
**Distribution over time of 2'-OMePS oligonucleotides and analysis of the duration of *in vivo* SSO-mediated skipping following a unique intravitreal injection**. (**a**) Histological analysis by confocal microscopy on retinal sections following a single intravitreal injection of 10 nmoles of (6-FAM)-m35ESE (left panel) or (6-FAM)-m36ESEsense (right panel). Note that while the fluorescent signals in the retina from eyes injected with the m35ESE or m35ESEsense oligonucleotide decreases overtime, a significant signal is still visible at 30 dpi of the SSO but not of the control oligonucleotide. Nuclei were labeled using DAPI (blue). GCL, ganglion cell layer; INL, inner nuclear layer; ONL, outer nuclear layer; OS, outer segment of photoreceptors. Scale bars: 50 µm. (**b**) Image of agarose gels of reverse transcription polymerase chain reaction products of *Cep290* mRNA extracted from untreated eyes (-) and eyes injected (+) with 1 μl of saline solution containing 10 nmoles of m35ESE SSO or m35ESEsense control oligonucleotide for 2, 12, or 30 days, respectively. The 297 bp PCR fragment (wild-type *Cep290* mRNA, *WT*) is detected in all retinas. An additional shorter 189 bp *PCR* product lacking exon 35 (*Δex35*) is detected in retina from all eyes injected with the SSO but not the control oligonucleotide from 2 to 30 dpi. (**c**) Relative expression of wild-type (*WT*, gray bars) and skipped *(Δex35*, black bars) *Cep290* mRNA in retina from uninjected (-) and injected (+; same conditions as in **b**) as determined by RT-qPCR using *Tbp* and *Hprt1* genes as reference. RT-qPCR analysis shows that the abundance of skipped *Cep290* mRNA decreases overtime but it is still measurable at 30 dpi. The expression level of *Cep290* wild-type mRNA was unchanged upon the injection of the m35ESEsense oligonucleotide. Conversely, it was significantly decreased in retina from eyes injected with the m35ESE SSO. Surprisingly, the level remained unchanged with the decrease of the mutant mRNA lacking exon 35 supporting the view of m35ESE-mediated RNA interference. The error bars represent the standard error of the mean derived from at least three eyes per experimental condition (23 mice in total, 1–23). Dpi, days postinjection.

To estimate the lifetime of SSOs and of mutant mRNAs after a unique iv injection, 10 nmoles of (6-FAM)-m35ESE SSO or (6-FAM)-m35ESEsense control oligonucleotides were injected. The distribution of the flurorescence and abundance of mutant mRNA in the retina were analyzed at days 2, 4, 8, 12, 18, and 30 postinjection (dpi) as described previously. We chose to use a SSO specific to exon 35 to preserve the reading frame and avoid degradation of mutant mRNA by nonsense-mediated mRNA decay mechanisms. Confocal imaging of retinal sections from eyes injected with the (6-FAM)-m35ESE SSO detected fluorescent signals in all retinal layers from 2 to 30 dpi (**Figure 3a**). In contrast, fluorescent signals arising from the (6-FAM)-m35ESEsense control oligonucleotide had disappeared at 12 dpi (**Figure 3a**), suggesting rapid clearance of oligonucleotides that do not find target. RT-PCR and RT-qPCR analysis of retinal mRNA detected the presence of *Cep290* mRNA lacking exon 35 as confirmed by Sanger sequencing (not shown), after the injection of the SSO but not the control oligonucleotide (**Figure 3b**,**c**). The abundance of mutant mRNA was maximum at 2 dpi and decreased gradually overtime but it was still measurable at 30 dpi. Surprisingly, that of the wild-type *Cep290* mRNA was significantly reduced at all analysis time points including 30 dpi (**Figure 3c**), suggesting that the m35ESE SSO which target an exonic splice enhancer might recognize the mature mRNA in the cytoplasm of retinal cells, and cause double-stranded RNA-mediated interference. To assess this hypothesis, we measured the wild-type and mutant mRNA abundance at 2, 6, and 10 dpi of 10 nmoles of the m22D SSO, which targets a part of intronic sequence, absent in the cytoplasm. We observed a moderate decrease of the wild-type mRNA abundance consistent with the production of a mutant mRNA at the expense of the *Cep290* pre-mRNA (**Supplementary Figure S4**). Together, these data are consistent with the hypothesis according to which SSOs recognizing exon-only sequences can be sequestrated by cytosolic mRNA molecules which are not subjected to nonsense-mediated mRNA decay.^16^ The high expression of *Cep290* in the retina^17^ further supports this hypothesis.

**Figure 4:**
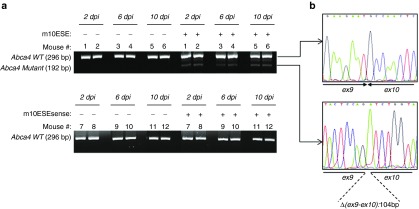
**SSO-mediated exon skipping occurs in photoreceptor cells**. Image of agarose gels and Sanger sequencing chromatograms of polymerase chain reaction (PCR) products obtained from reverse transcribed retinal mRNA from untreated eyes (-) and eyes injected (+) with 1 μl of saline solution containing 10 nmoles of *Abca4* m10ESE SSO or m10ESEsense control oligonucleotide for 2, 6, or 10 days, respectively. The 296 bp PCR fragment corresponding to the wild-type *Abca4* mRNA is detected in all retinas. An additional shorter 192 bp PCR product lacking a 104 bp sequence partly overlapping exons 9 and 10 (Δ(ex9-ex10)) is detected in retina from eyes injected with the SSO but not the control oligonucleotide from 2 to 10 dpi. Samples from 24 eyes from 12 mice (1–12) are shown. Dpi, days postinjection.

The subcellular expression of *Cep290* mRNA in the mouse retina has not been documented to our knowledge. In the zebrafish, *in situ* hybridization analysis has shown that the gene is expressed in all retina cell layers, including the ganglion cell layer, the inner nuclear layer and the photoreceptors cell layer.^18^ To assess whether photoreceptor cells contribute to the pool of switched mRNA, we designed a SSO specific to the photoreceptor-specific *Abca4* gene.^19^ Intravitreal injection of 10 nmoles of the *Abca4*-specific SSO (m10ESE) but not of its sense version (m10ESEsense) resulted in maintained (≥10 dpi) *Abca4*-specific splicing modification, as evidenced by agarose gel electrophoresis and Sanger sequencing of RT-PCR products (**Figure 4**). This demonstrates that splicing can be manipulated in photoreceptor cells following a single iv injection of 2'-OMePS SSO.

Finally, to evaluate the ocular toxicity of 2'-OMePS oligonucleotides, we injected 10 nmoles of the m35ESEsense oligonucleotide and we examined the retinal structure integrity, photoreceptor survival, and inflammatory response at 2, 12, and 30 dpi. Retinas from injected eyes were compared to retinas from uninjected contralateral eyes and retinas from 22-day-old *rd10* mice eyes (**Supplementary Figure S5**). The *rd10* mouse carries a spontaneous missense point mutation in *Pde6b* responsible for progressive retinal outer nuclear layer degeneration beginning at postnatal day 16.^20^ Hematoxylin-eosin staining, terminal deoxynucleotidyl transferase-mediated dUTP nick end labeling (TUNEL), and glial fibrilary acidic protein labeling on histological sections of 22-day-old *rd10* mice evidenced decreased thickness of the outer nuclear layer, apoptotic cells, and positive glial fibrilary acidic protein astrocytes, respectively (**Supplementary Figure S5**). In contrast, the retinal structure, TUNEL labeling, and glial fibrilary acidic protein staining of uninjected and injected eyes supported absence of retinal cell death and of inflammatory cell infiltration, respectively (**Supplementary Figure S5**). The transitory TUNEL labeling observed at 2 dpi in treated eyes likely arose from dUTP nick end labeling of the free 3'-end of the oligonucleotide (**Supplementary Figure S6**). The absence of deterioration of the retinal structure and the correlation between the clearance of the TUNEL labeling and that of the m35ESEsense oligonucleotide (**Figure 3**) strongly support this hypothesis.

## Discussion

AAV-based gene therapy developments are ongoing worldwide to treat a wide range of inherited retinal diseases since the report of safety and efficacy in phase 1/2 clinical trials of a *RPE65* gene augmentation therapy, by subretinal delivery of AAVs carrying the wild-type *RPE65* cDNA.^2,3,4^ A severe limitation of this approach however is the limited packaging capacity (~4.9 Kb) of AAV vectors. In addition to *CEP290*, several other of the most frequently mutated inherited retinal dystrophy genes, *e.g.*, *ABCA4, EYS*, and *USH2A* have a cDNA size way exceeding this limit and hence are not amenable for AAV-based gene therapy. Other vectors (*e.g.*, lentiviruses, adenoviruses) with a larger cargo capacity have a limited tropism for photoreceptor cells, and may produce insertional mutagenesis by integration into the host genome. Unintended gene overexpression and/or imbalanced expression of splicing isoforms are other severe limitations of gene augmentation therapy, especially when the gene, like *CEP290*, is transcribed into multiple isoforms and/or contribute to large protein complexes which stoichiometry has to be respected.^21,22^ Rescuing aberrant pre-mRNA processing rather than supplementing a healthy cDNA copy of a gene that is mutated is an interesting therapeutic alternative. It is ideally suited to overcome both size limitations and unintended gene overexpression and imbalanced expression of splicing isoforms by maintaining endogenous transcriptional regulation of the target gene; acting at the pre-mRNA level allows correcting the genetic defect where and when the gene is expressed.

Strategies have been developed which allow the modification of splice patterns of genes to bypass mutations that silence consensus splice sites and/or activate cryptic splice sites and hence promote exonic exclusion and/or intronic retention. Splicing can be altered by masking splice sites, or by targeting regulatory sequences to promote or to block splicing.^23^ Drugs, isoform-specific antibodies, trans-splicing approaches, RNA interference, and antisense oligonucleotides have been investigated for their ability to modify splicing in various diseases.^23,24,25,26,27^ Compared to most other molecules, nuclease-protected phosphorothioate antisense oligonucleotides are of easy design^28^ and inexpensive. In addition, they can be widely used to restore wild-type genetic configuration by allowing skipping of stray pseudoexons such as the one which arises from the splicing of pre-mRNAs harboring the recurrent LCA-causing *CEP290* c.2991+1655A>G mutations.^12,29^

Current ongoing trials in patients affected with Duchenne muscular dystrophy have shown that systemic delivery of 2′-OMePS SSOs on a short-term basis is well tolerated.^30,31,32^ Systemic administration of 2′-OMePS SSOs to correct the abnormal splicing resulting from the *CEP290* c.2991+1655A>G is hampered by the very efficient blood–retina barrier. Likewise the impermeable nature of the cornea hampers non-invasive topical delivery.^14^ Here, we present data which demonstrate that iv administration of 2'-OMePS oligonucleotides allows alteration of *Cep290* splicing in retinal cells, including photoreceptors as shown by successful alteration of *Abca4* splicing using the same approach. We were able to detect both fluorescent signals arising from 6-FAM-labeled antisense oligonucleotides and skipped mRNA 1 month after a single iv injection. Interestingly, it has been previously reported that fluorescent signals in 6-FAM-injected eyes rat completely disappeared at 24 hours after iv injection.^33^ The persistence over several weeks of fluorescent signals in eyes that received the labeled 2'-OMePS SSO indicates that the fluorescence is associated with the oligonucleotide itself rather than the fluorescent molecule that was used for labeling. In addition, we observed that the persistence of the fluorescence arising from 6-FAM sense oligonucleotides was strikingly reduced (lost between 2 and 12 dpi) compared to that arising from 6-FAM antisense oligonucleotides (still present at 30 dpi). This indicates that oligonucleotides which do not find target are rapidly cleared. This observation along with the progressive decrease overtime of skipped mRNA abundance in eyes injected with antisense oligonucleotides suggest that the detection of skipped mRNA at 1 month reflects active splice switching rather than mRNA stability.

In this study, we analyzed the retina no longer than 1 month after injection. Other studies have however shown that RNA-interfering phosphorothioate antisense oligonucleotides (PS-AON) could be detected in the retina of rodent and nonprimate humans as full-length oligonucleotide at least for 6 weeks and as N-1 for up to 12 weeks after injection.^33,34^ Antisense oligonucleotides with modified phosphorothioate backbones (2'-OMePS SSO) have increased stability compared to PS-AONs.^35^ Hence, it is likely that the effect of a single iv injection should exceed 4 weeks.

CEP290 is a dynamic protein which can incorporate into preassembled mutant cilia transition zone. Providing functional CEP290 should allow restoring visual function as long as there are spared photoreceptors. LCA patients with *CEP290* mutations have a neonatal-onset severe panretinal loss of photoreceptors.^10,36^ While patients retain a central retinal zone of photoreceptors and an intact visual cortex for up to three decades, it has been suggested from gene augmentation therapeutic trials that the earliest the treatment, the better the effect.^36,37,38^ Conversely, to subretinal administration which is limited central retina to avoid severe retinal detachment, intravitreal injections of 2'-OMePS seems suited for panretinal treatment. Indeed, following an iv injection of 6-FAM 2'-OMePS SSO, we were able to detect fluorescent signals in all retinal cell layers from the centre to the far periphery supporting the view that the oligonucleotides were able to distribute widely in the target tissue.

An important concern before efficacy study of iv injection-based SSO-mediated therapy is to investigate the intraocular safety. Here, we report no adverse event at 1 month following a single iv injection of 10 nmoles of oligonucleotide in the mouse. Although this study is limited and 2'-OMePS SSO chemistry differs slightly for PS-AON, it is worth remembering that repeated iv injections of Vitravene, a 22-mer RNA interfering PS-AON has been approved by the FDA to treat CMV-induced retinitis in immune-compromised individuals.^39^ In addition, it is worth noting that here the duration of the SSO effect over at least 1 month is consistent with iv injection-based treatment, even in children. Indeed, although iv injection is invasive, it is a very well-established mode of delivery of therapeutic molecules both in infants with acute retinal diseases and adults with acute or chronic retinal diseases.^40,41^

The advantages of SSO-based therapy over viral-based gene augmentation protocols give strong justification to the development of oligotherapy as a mean to bypass the *CEP290* c.2991+1655A>G mutation. SSO therapy is not limited to this important therapeutic target or to other deep intronic mutations. The vast majority of *CEP290* mutations introduce a premature termination codon in the mRNA. In addition to isolated early-onset severe retinal degenerations, these mutations cause a wide spectrum of multisystemic ciliopathies.^8^ Very recently, basal exon skipping (BES), a genetic mechanism through which deleterious mutations may be partially compensated for, has been reported to occur spontaneously at low levels in all *CEP290* patient fibroblasts. The gradation of CEP290 dysfunctions likely correlates with the amount of near-full length protein retaining all or some of the full-length protein functionality that can be supplied to the cell through this mechanism.^42,43^ The report of selective exclusion of an in-frame *CEP290* exon with a premature termination codon through nonsense-associated altered splicing^44,45,46^ in an individual with unexpectedly mild retinal disease^47^ is quite consistent with the hypothesis. Interestingly, SSOs are ideally suited to enhance BES of mutant exons that begin and end in the same reading frame and which do not code for a crucial protein domain, to increase the abundance of minimally shortened mRNA and hence of near-full length functional CEP290 protein. What holds true for CEP290 could be true for other retinal proteins. Evidence of splicing alteration using intravitreal administration of SSO could open avenues to oligotherapy of a wide range of dysfunction affecting retinal cell wherever their geolocalization in the tissue.

The study we report here was limited by the use of wild-type animals. Availability of a spontaneous animal model of severe retinal dystrophy due to an intronic *CEP290* mutation, the *rdAc* cat, will hopefully help in making the proof of concept of therapeutic efficiency of iv injection based SSO-mediated therapy.

## Materials and methods

*Design of SSOs and control oligonucleotides (ONs).* SSOs were designed from the wild-type *mus musculus Cep290* (UCSC accession number uc011xmr.1) and *AbcA4* (UCSC accession number uc008rel.1) sequences using the m-fold and ESEfinder^48^ programs available online at http://mfold.rna.albany.edu/ and http://rulai.cshl.edu/cgi-bin/tools/ESE3/esefinder.cgi, respectively. SSOs were designed to target ESE and donor-splice site sequences of *Cep290* exon 22 and 35 and an ESE sequence of *Abca4* exon 10, respectively. Sense versions of SSOs (control ONs) targeting *Cep290* and *Abca4* ESE sequences were designed to control the specificity of SSOs. SSOs and control ONs were synthetized as fluorescently labeled (6-FAM) and unlabeled 2'-O-methyl phosphorothioate oligonucleotides by Sigma-Aldrich (St Quentin Fallavier, France).

The positions of SSOs and control oligonucleotides are represented in **Figure 1**. The sequences (5′-3′) were as following: m22ESE gaugacgaaucacugcaaac; m22ESEsense guuugcagugauucgucauc; m22D guuuucaaaauauaaauaccuuagguauuc; m35ESE caugaaggucuuccucaugc; m35ESEsense gcaugaggaagaccuucaug; m35D guucucagaaucuuaccugagcug; m10ESE caaagaaguaccagaucuggggcccuac; m10ESEsense guagggccccagaucugguacuucuuug.

*Intravitreal injection of SSOs and control ONs.* All animal experiments adhered to the French regulation statement for the use of animals in ophthalmic and vision research. Eight-week-old C57BL/6J mice were used for these experiments. Prior to intravitreal injection, the animals were anesthetized by intramuscular injection of mixture solution of ketamine (100 mg/kg) and xylazine (10 mg/kg). The left pupil was dilated by applying one drop of a mydriatic mix containing 10% phenylephrine and 0.5% tropicamide. A 30-gauge needle was used to make an initial puncture of the sclera. 1 µl of saline solution (NaCl 9 g/l, pH = 8.7) containing 1, 5, or 10 nmoles of (6-FAM)-m22D, (6-FAM)-m35ESE SSOs, or (6-FAM)-m35ESEsense oligonucleotide, respectively was injected through this hole using a 33-gauge needle attached to a 5 μl Hamilton syringe, under a binocular magnifier. The needle was kept in the vitreous cavity for about 20 seconds, then withdrawn gently and an antibiotic ointment was applied to prevent infection. The right eyes were left noninjected and used as controls. To assess splice alteration in photoreceptor cells, 10 nmoles of the m10ESE SSO and m10ESEsense oligonucleotide were respectively injected using the same procedure. Injected and contralateral untreated eyes were enucleated at variable time points from 2 to 30 dpi. Sampled eyes were either fixed in PFA 4%, washed in PBS and embedded in paraffin for histological analyses; or dissected to recover retinal RNA; as described below. Between two and five animals were used for each experimental setup.

*RNA extraction and cDNA synthesis.* Total RNA from treated and untreated NIH3T3 cells and mouse retinas was extracted using the RNeasy Mini Kit (Qiagen, Courtaboeuf, France) according to manufacturer's protocol. All samples were DNase treated by the RNase-free DNase set (Qiagen). Concentration and purity of total RNA was determined using the Nanodrop-1000 spectrophotometer (Thermo Scientific, Illkirch, France) before storage at −80 °C. First-stranded cDNA synthesis was performed from 500 ng of total RNA extracted using Verso cDNA kit (Thermo Scientific) with random hexamer:anchored oligo(dT) primers at a 3:1 (vol:vol) ratio according to the manufacturer's instructions. A non-RT reaction (without enzyme) for one sample was prepared to serve as control in RT-qPCR experiments.

*RT-PCR.* To assess the efficiency of SSO-mediated exon skipping, cDNAs (5 µl) were amplified in 50 µl of 1× Phusion HF buffer containing 5 mmol/l dNTPs (Thermo Scientific), 0.02 units of Phusion High-Fidelity DNA polymerase (Thermo Scientific), and 10 µmol/l of each primer *Cep290(ex21)* forward, 5′-gaccaccttgagaaggaaac-3′ and *Cep290(ex23)* reverse, 5′-catcctgctcagcttgatc-3′ or *Cep290(ex34)* forward, 5′-cccaccaaactattgccaac-3′ and *Cep290(ex36)* reverse, 5′-gagagtcatcttgttctgctac-3′—or - *Abca4(ex9)* forward, 5′-tgatccagagcctggagtcaa-3′ and *Abca4(ex11)* reverse, 5′-ttcttctccgagctgcctatt-3′. PCRs were carried out on a 2720 Thermal Cycler (Applied Biosystems, Courtaboeuf, France) under the following conditions: initial denaturation at 98 °C for 5 minutes, followed by 30 cycles of 10 seconds denaturation at 98 °C, 30 seconds annealing at 60 °C and 30 seconds extension at 72 °C. The *PCR* products were separated (20 µl) by electrophoresis in a 3% agarose gel stained with ethidium bromide and visualized under UV lights. No template control reactions were used as negative control. The final confirmation of identity of these products was carried out by Sanger sequencing to establish that the correct and expected exon junctions have been maintained.

*Real-time quantitative PCR (RT-qPCR).* To measure the level of expression of mouse *Cep290* mRNAs, the wild-type and mutant transcripts were amplified as 102 and 75 bp fragments (exon 22 skipping), respectively; or the wild-type and mutant transcripts were amplified as 100 and 62 bp fragments (exon 35 skipping), respectively. The mouse TATA boxbinding protein mRNA (*Tbp,* NM_013684.3), the mouse β-2-microglobulin mRNAs (*B2m*, NM_009735.3), the mouse β-glucuronidase mRNAs (*Gusb*, NM_010368.1), the mouse hypoxanthine phosphoribosyltransferase 1 mRNA (*Hprt1,* NM_013556.2), and the mouse peptidylprolyl isomerase A mRNA (*Ppia*, NM_008907.1) were used for normalization. The mouse albumin gene (*Alb*, NM_009654.3) was used to control the noncontamination of cDNAs by genomic DNA. Primers were designed using the Oligo Primer Analysis Software v.7 available at http://www.oligo.net. The specificity of primer pairs to PCR template sequences was checked against the NCBI database using the Primer-BLAST software (http://www.ncbi.nlm.nih.gov/tools/primer-blast). Primer sequences were as follows: *Cep290ex22wt* forward, 5′-tgactgctaagtacagggacatcttg-3′; *Cep290ex22wt* reverse, 5′-aggagatgttttcacactccaggt-3′; *Cep290ex22mt* forward, 5′-ctggccccagttgtaatttgtga-3′; *Cep290ex22mt* reverse, 5′-ctgttcccaggcttgttcaatagt-3′; *Cep290ex35wt* forward, 5′-tgactgctaagtacagggacatcttg-3′; *Cep290ex35wt* reverse, 5′-aggagatgttttcacactccaggt-3′; *Cep290ex35mt* forward, 5′-ctggccccagttgtaatttgtga-3′; *Cep290ex35mt* reverse, 5′-ctgttcccaggcttgttcaatagt-3′. Reference genes *Tbp* forward, 5′-tgacctaaagaccattgcacttcgt-3′; *Tbp* reverse, 5′-ctgcagcaaatcgcttggga-3′; *B2m* forward, 5′-cctgtatgctatccagaaaacccct-3′; *B2m* reverse 5′-cgtagcagttcagtatgttcggctt-3′; *Gusb* forward, 5′-ctgcggttgtgatgtggtctgt-3′; *Gusb* reverse, 5′-tgtgggtgatcagcgtcttaaagt-3′; *Hprt1* forward, 5′-gttggatacaggccagactttgtt-3′; *Hprt1* reverse, 5′-aaacgtgattcaaatccctgaagta-3′; *Ppia* forward, 5′-ccaaacacaaacggttcccagt-3′; *Ppia* reverse, 5′-gcttgccatccagccattca-3′; *Alb* forward 5′-gggacagtgagtacccagacatcta-3′; *Alb* reverse 5′-ccagacttggtgttggatgctt-3′. cDNAs (5 μl of a 1:25 dilution in nuclease-free water) were subjected to real-time PCR amplification in a buffer (20 μl) containing SYBR GREEN PCR Master Mix (Applied Biosystems) and 300 nmol/l of forward and reverse primers, on a MasterCycler epgradients Realplex2 (Eppendorf, Hamburg, Germany) under the following conditions: Taq polymerase activation and initial denaturation at 95 °C for 10 minutes, followed by 50 cycles for 15 seconds at 95 °C, and 1 minute at 62 °C. The specificity of amplification products was determined from melting curve analysis performed at the end of each run using a cycle at 95 °C for 15 seconds, 60 °C for 15 seconds, and 95 °C for 15 seconds. Data were analyzed using the Realplex software (Eppendorf). For each cDNA sample, the mean of quantification cycle (*C*_q_) values was calculated from triplicates (SD <0.5 *C*_q_). *Cep290* expression levels were normalized to the “normalization factor” obtained from the geNorm v3.5 software for Microsoft Excel which uses the most stable reference genes and amplification efficiency estimates calculated for each primer pair using fourfold serial dilution curves (1:5, 1:25, 1:125, and 1:625). No reverse transcriptase (non-RT), no template control reactions, and noncontamination of cDNAs by genomic DNA (*Alb*) were used as negative controls in each run (*C*_q_ values no template control = undetermined, non-RT >35 and *Alb* >35). The quantitative data are the means (±standard error of the mean) of three or more independent experiments and these are presented as ratio among values for individual mRNAs.

*Retinal distribution of fluorescence following a unique intravitreal injection of SSOs or control oligonucleotide.* Seven-micron serial sections of tissues were prepared from paraffined treated and untreated eyes. To evaluate the dissemination of (6-FAM)-m22D, (6-FAM)-m35ESE SSOs and (6-FAM)-m35ESEsense oligonucleotide through the retina, the tissue sections were mounted onto glass slides with Prolong gold antifade mounting media containing 4′,6′-diamidino-2-phenylindole (DAPI) (Invitrogen) to stain nuclei and examined by confocal microscopy (ZEISS LSM700). Images were generated using the ImageJ software.

*Statistical analysis.* The statistical significance of the difference between three or more means was determined using a Mann-Whitney test. Statistical analysis was performed using the Biostatgv program available online at http://biostatgv.sentiweb.fr. *P values < 0.05 were considered significant.

**SUPPLEMENTARY MATERIAL** **Figure S1.** Optimization of NIH3T3 transfections. **Figure S2.** SSO-mediated skipping of *Cep290* exons 22 and 35 in NIH3T3 cells. **Figure S3.** Panretinal distribution of SSO following a unique intravitreal injection. **Figure S4.** Kinetic analysis of SSO-mediated *Cep290* exon skipping following a single intravitreal injection in the C57BL/6J wildtype mouse. **Figure S5.** Toxicological evaluation of the retina. **Figure S6.** TUNEL-staining of oligonucleotides.
