# Supplementary material for: Intravitreal Injection of Splice-switching Oligonucleotides to Manipulate Splicing in Retinal Cells
Source: Mol Ther Nucleic Acids. 2015 Sep 1;4(9):e250–. doi: 10.1038/mtna.2015.24 (PMC4877449; doi:10.1038/mtna.2015.24)
Supplement: Supplementary Figures [file mtna201524x1.doc]

**Supplementary Material**

**Intravitreal injection of splice-switching oligonucleotides to manipulate splicing in retinal cells.** Xavier Gérard^1^, Isabelle Perrault^1^, Arnold Munnich^1^, Josseline Kaplan^1^, Jean-Michel Rozet^1^,*

^1^ Laboratory of Genetics in Ophthalmology, Inserm UMR1163, Institut *Imagine*, Université Paris Descartes Sorbonne Paris Cité, Hôpital Necker, 75015 Paris, France.

* Address correspondence to JMR LGO, Institut Imagine, 24 boulevard du Montparnasse, 75015 Paris, France. Tel: + 33 (0)1.42.75.43.06; Email: [jean-michel.rozet@inserm.fr](mailto:jean-michel.rozet@inserm.fr)

**Materials and Methods**

***Cell culture and transfection of SSOs and control oligonucleotides***

Mouse fibroblasts (NIH3T3) were obtained from the American Type Culture Collection (Rockville, Md.). NIH3T3 cells (<15 passages) were used in our studies and were cultured in a standard medium consisting of DMEM (Invitrogen, St Aubin, France) containing 10% FCS, 50 U/ml penicillin and 50 mg/ml streptomycin (Invitrogen). The day before transfection, cells were either plated in six-well plates. Cells at 80 % of confluence were transfected with 150, 300, 450 and 600 nM of oligonucleotide in Opti-MEM medium (Invitrogen) using 2.5 µl of Lipofectomine2000 reagent (Invitrogen) per microgram of oligonucleotide. After 4 hours of incubation at 37 °C, the transfection medium was replaced by fresh culture medium. Cells were harvested for mRNA analysis between 24 and 72 hours.

***Transfection efficiency***

To control that transfection efficiencies were independent of oligonucleotide sequences, NIH3T3 cells were seeded on glass coverslips in 12-well plates, 24 hours before transfection. The cells were transfected as described previously using 150 nM of the (6-FAM)-m22D, (6-FAM)-m35D SSOs and the (6-FAM)-m22ESEsense oligonucleotide, respectively. After 4 hours, cells were fixed with PFA 4% (15 minutes at room temperature) and washed twice in PBS. Nuclei were labeled using a mounting media containing 4′,6-diamidino-2-phenylindole (DAPI) (ProLong Gold antifade reagent with DAPI; Invitrogen). Immunofluorescence images were obtained from a ZEISS LSM700 confocal microscope (Carl Zeiss, Germany). The final images were generated using ImageJ (National Institutes of Health, Bethesda, MA). Percentages of fluorescent cells were calculated from three independent experiments for each oligonucleotide transfection (n > 100 counted cells for each transfection).

***Immunoprecipitation and Western blot analysis***

Untreated and NIH3T3 cells transfected with 150 nM of m22ESE were harvested 24 hours after treatment and lysed in RIPA buffer (Sigma-Aldrich) containing complete protease inhibitor cocktail (1%; Sigma-Aldrich) on ice for 1 hour with repeated mixing. Lysis was accomplished by 15 seconds of sonication on ice (VibraCell 72434; Bioblock Scientific, Illkirch, France) and the lysates were centrifuged (13,000 rpm at 4 °C for 10 minutes). 800 µg of protein extracts were analyzed by immunoprecipitation (IP) using µMacs Separation Columns and µMacs Protein G Microbeads (Miltenyi Biotec, Paris, France) with a rabbit polyclonal anti-CEP290 (1:100; Novus Biologicals, Littletown, CO), according to supplier’s recommendations. Immunoprecipitated proteins, on the one hand, and 150 µg of initial protein extracts resuspended in LDS sample buffer 1X (Life Technologies, St Aubin, France) with 10% β-mercaptoéthanol, on the other hand, were heated at 90 °C for 10 min and loaded on a 4-15% Mini-PROTEAN TGX precast polyacrylamide gels (BioRad, Marnes-la-Coquette, France). After electrophoresis, proteins were transferred to a 0.2µm PVDF membrane using the Trans-Blot Turbo transfer system (BioRad) which was probed with the following primary antibody: rabbit polyclonal anti-human Cep290 (1:1800; Novus Biologicals, Littletown, CO) and secondary antibody: goat anti-rabbit IgG-HRP (1:5,000; Abcam, Paris, France). Blots were revealed with the use of SuperSignal®West Dura Extended Duration Substrate (Thermo Scientiﬁc) and ChemiDoc XRS+ Imaging System (Bio-Rad). Western blot images were acquired and analyzed with the Image Lab Software 3.0.1 build 18 (Bio-Rad).

***Histological and immunohistological analysis of the retinal structure, apoptosis and inflammation following a unique intravitreal injection of 2’-OMePS* oligonucleotide**

To assess the ocular toxicity of *2’-OMePS* oligonucleotide*,* retinas from untreated eyes were compared to retinas for eyes treated with 10 nmoles of (6-FAM)-m35ESEsense oligonucleotide for 2, 12 and 30 days. A retina from a *rd10* C57BL/6J mouse aged 22 days was used as a positive control of retinal degeneration. The retinal structure was assessed under a light microscope (LEICA DMR, Germany) from hematoxylin/eosin stained retinal sections. Apoptotic cells in tissue sections were detected using the DeadEnd™ Colorimetric TUNEL System (Promega, Charbonnières-les-bains, France) according to the manufacturer’s instructions. Tissues sections were examined with a light microscope (LEICA DMR, Germany). Inflammation was assessed on retinal sections by immunohistochemistry using a rabbit polyclonal anti-GFAP (overnight at 4°C; 1:400; Dako, Les Ulis, France). Primary anti-GFAP antibody was detected by incubating the section for 1h at room temperature using a goat anti-rabbit IgG (1:200, Alexa Fluor 568; Molecular Probes, St Aubin, France) secondary antibody. Prolong gold antifade mounting media (Invitrogen) was used to mount the sections. GFAP staining was detected using confocal microscopy (ZEISS LSM700). All images were generated using the ImageJ software.

***Other experiments***

Details of RNA extraction and cDNA synthesis, RT-PCR and RT-qPCR analysis, and protocol of 2'-OMePS oligonucleotides injection can be found in the Materials and Methods section of the main manuscript.

**Supplementary figures:**


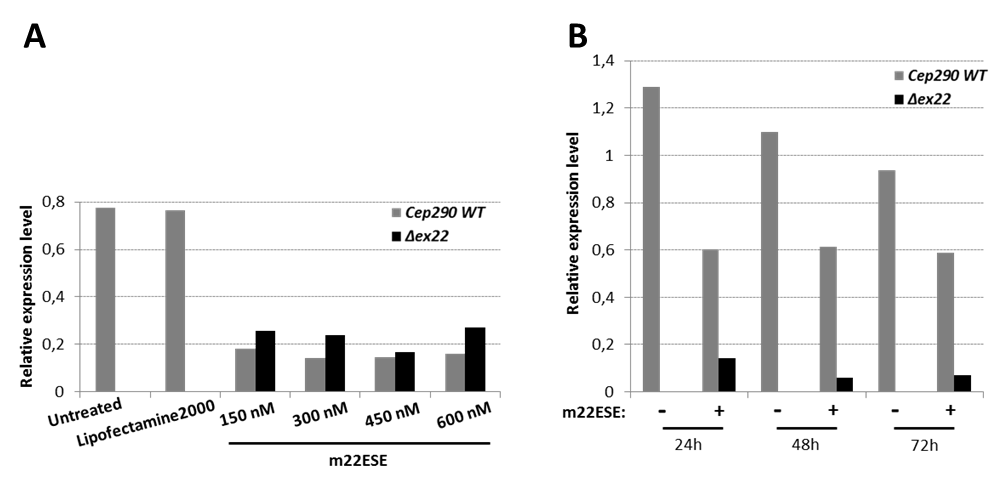


**Fig. S1. Optimization of NIH3T3 transfections.** (**A**) Relative expression of wildtype (*WT*, gray bars) and skipped *(Δex22*, black bars) in cells transfected with variable doses (150, 300, 450 or 600 nM) of m22ESE SSO for 24 hours and untreated NIH3T3 cells, as determined by RT-qPCR with *Gusb* and *Ppia* genes as reference. *Cep290* expression levels in untreated cells and cells transfected with lipofectamine2000 alone are in the same range, suggesting that the transfection reagent does not alter *Cep290* expression in NIH3T3 cells. The striking reduction in expression of the wildtype mRNA and the apparition of a mutant mRNA observed in cells treated with the complex lipofectamine2000 + SSO is consistent with efficient SSO mediated-skipping. The lowest efficient SSO concentration (150 nM) was selected for further analyses in NIH3T3 cells. (**B**) Relative expression of wildtype (*WT*, gray bars) and skipped *(Δex22*, black bars) in NIH3T3 cells treated with 150 nM of the m22ESE SSO (+) and untreated (-) cells, as determined by RT-qPCR with *Gusb* and *Ppia* genes as reference. The relative abundance of the skipped *Δex22* mRNA is maximal at 24h following the transfection; this time point has been selected for further analyses in NIH3T3 cells.


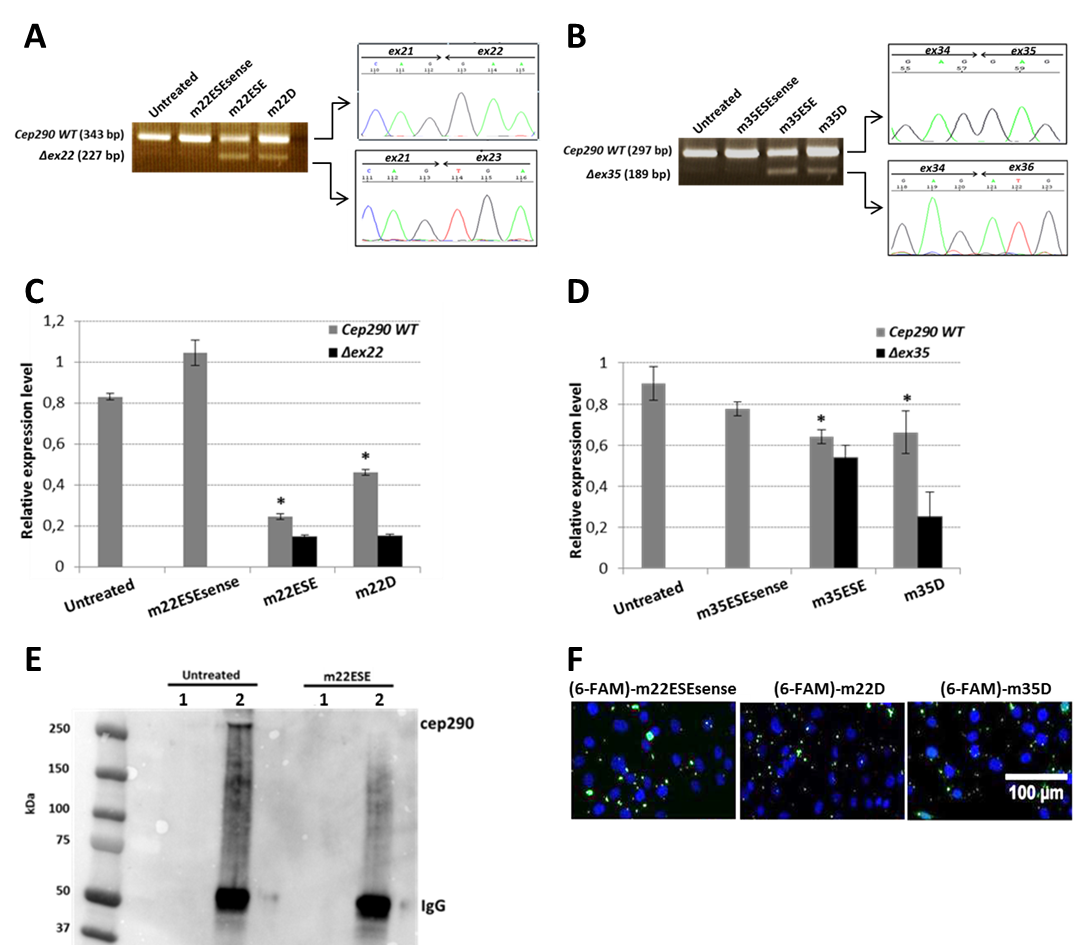


**Fig. S2. SSO-mediated skipping of *Cep290* exons 22 and 35 in NIH3T3 cells.** (**A, B**) Image of agarose gels and Sanger sequencing chromatograms of *PCR* products obtained from reverse transcribed mRNA from untreated cells or cells treated for 24 hours with 150 nM of SSOs (m22ESE, m22D, m35ESE, m35D) and control oligonucleotide (m22ESEsense, m35ESEsense), respectively. Unique PCR fragments corresponding to the wildtype *Cep290* exon 22 (343 bp) or 35 (297 pb) mRNA are detected in all samples. Upon transfection with SSOs but not control oligonucleotides, additional shorter *PCR* products are detected which sequence show absence of exon 22 (227 bp, *Δex22)* and exon 35 (189 bp, *Δex35)*, respectively. (**C, D**) Relative expression of wildtype (*WT*, gray bars) and skipped *(Δex22 or Δex35*, black bars) *Cep290* mRNA in treated and untreated NIH3T3 cells (same conditions as in A, B) as determined by RT-qPCR using *Gusb* and *Ppia* genes as reference. Transfection using the SSOs but not the control oligonucleotide targeting exon 22 and 35 resulted in a significant decrease in the wildtype *Cep290* mRNA abundance and the apparition of mutant mRNA lacking exon 22 and 35, respectively. The error bars represent the standard error of the mean (SEM) derived from three independent experiments (**p < 0.05*). (**E**) Western blot analysis of proteins extracted from untreated NIH3T3 cells and cells treated with 150 nM of the m22ESE SSO for 24h. Using a rabbit polyclonal antibody raised against the C-terminal residues, Cep290 could be detected in immune-precipitates (2) prepared from 800 μg of total protein but not from 150 μg of total protein extracts (1). (**F**) Confocal microscopy analysis of NIH3T3 cells transfected for 4h with 150 nM of the (6-FAM)-m22D and (6-FAM)-m35D SSOs, and the (6-FAM)-m22ESEsense oligonucleotide, respectively. Transfection efficiencies (calculated from three independent experiments and n > 100 cells) were all > 90 %, supporting sequence-specific SSO-mediated skipping. Nuclei were labeled using DAPI (blue). Scale bar 100 µM.


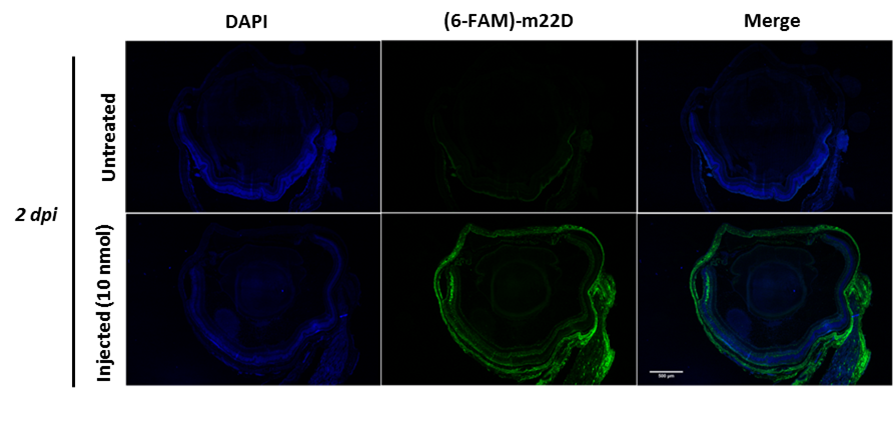


**Fig. S3. Panretinal distribution of SSO following a unique intravitreal injection.** Confocal microscopic presentation of large retinal sections from the untreated eye versus the injected eye (10 nmoles of (6-FAM)-m22D 2'-OMePS SSO) at 2 dpi. Nuclei were labelled using DAPI (blue). Dpi: days post-injection. Scale bar: 500 µM.


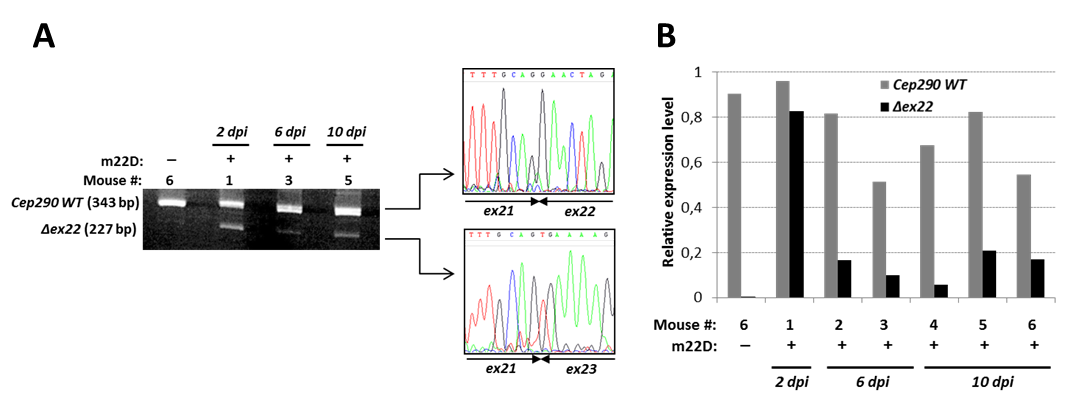


**Fig. S4. Kinetic analysis of SSO-mediated *Cep290* exon skipping following a single intravitreal injection in the C57BL/6J wildtype mouse.** (**A**) Image of agarose gels and Sanger sequencing chromatograms of *PCR* products obtained from reverse transcribed retinal mRNA from untreated eyes (-) and eyes injected (+) with 1 μl of saline solution containing 10 nmoles of m22D SSO for 2, 6 or 10 days, respectively. The 343 bp PCR fragment corresponding to the wildtype *Cep290* mRNA is detected in all retinas. An additional shorter 227 bp *PCR* product which sequence demonstrate skipping of exon 22 is detected in retina from all eyes injected with the SSO but not the control oligonucleotide. (**B**) Relative expression of wildtype (*WT*, gray bars) and skipped *(Δex22*, black bars) *Cep290* mRNA in retina from uninjected (-) and injected (+; same conditions as in A) as determined by RT-qPCR using *Tbp* and *Hprt1* genes as reference. A moderate decrease in the wildtype *Cep290* mRNA abundance and the apparition of mutant mRNA lacking the exon 22 were measured from 2 to 10 dpi. Samples from 7 eyes from 6 mice (1-6) are shown. Dpi: days post-injection.


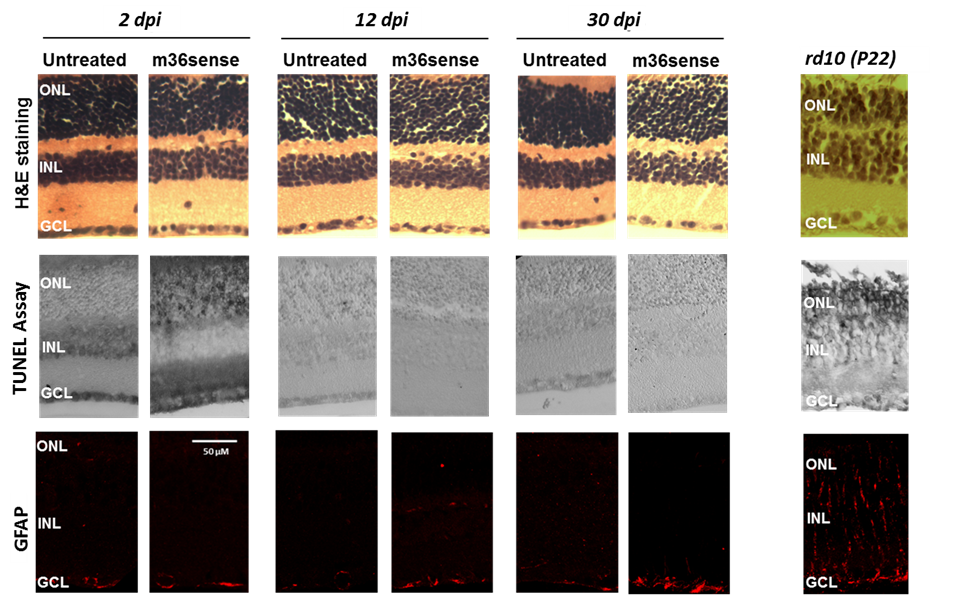


**Fig. S5. Toxicological evaluation of the retina.** To assess ocular toxicology, retina from eyes treated with 10 moles of m35ESEsense oligonucleotide for 2, 12 and 30 days were analyzed for morphological changes cell death and inflammation by hematoxylin and eosin (H&E) staining (line 1), colorimetric TUNEL assay (line 2) and immunolabeling of GFAP (red; line 3), respectively. Retinas from treated eyes were compared to retina from uninjected eyes and from 22 day-old (P22) *rd10* mouse eyes. A significant reduction of the outer nuclear layer and a strong staining of the GFAP protein are observed in the retina from *P22 rd10* mouse eyes but not in the retina from uninjected and injected eyes. As expected, a strong TUNEL-staining is noted in the retina from *P22 rd10* mouse eyes but not in the retina from uninjected wildtype eyes. In the retina from injected wildtype eyes a strong TUNEL-staining is noted at 2 dpi but not at later analysis time points, suggesting TUNEL-staining of the oligonucleotides (Fig. S7). Same eyes were used for all these histological analyses shown, using optical and confocal microscopy. Dpi: days post-injection. GCL: ganglion cell layer; INL: inner nuclear layer; ONL: outer nuclear layer. Scale bar: 50 µM.


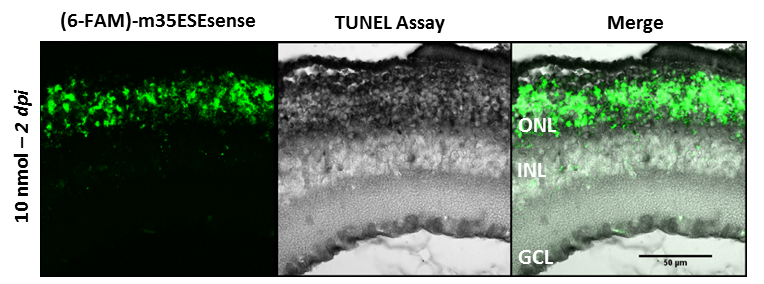


**Fig. S6. TUNEL-staining of oligonucleotides.** Histological analysis by confocal microscopy of retinal section at 2 dpi following a single intravitreal injection of 10 nmoles of (6-FAM)-m35ESEsense, showing an overlap of TUNEL-staining and fluorescent signal from the oligonucleotide. Biotinylated nucleotides have likely reacted with the free 3’-OH end of oligonucleotides as if it were fragmented DNA. Dpi: days post-injection. GCL: ganglion cell layer; INL: inner nuclear layer; ONL: outer nuclear layer. Scale bar: 50 µM.
